# Disease burden and attributable risk factors for chronic obstructive pulmonary disease in China, Japan, and South Korea: trends for 1990 to 2021 period and predictions for 2031

**DOI:** 10.3389/fmed.2025.1609322

**Published:** 2025-09-11

**Authors:** Yifan Wang, Jingwen Zhu, Shaoqiang Wang, Jihong Zhou

**Affiliations:** ^1^The Seventh Clinical College of Guangzhou University of Chinese Medicine, Shenzhen, China; ^2^Shenzhen Bao’an Chinese Medicine Hospital, Shenzhen, China; ^3^The Fourth Clinical Medical College of Guangzhou University of Chinese Medicine, Shenzhen, China; ^4^Shenzhen Traditional Chinese Medicine Hospital, Shenzhen, China; ^5^School of Information and Control Engineering, Qingdao University of Technology, Qingdao, China

**Keywords:** East Asian, GBD database, chronic obstructive pulmonary disease, disease burden, ageperiod-cohort analysis, Bayesian age-period-cohort analysis

## Abstract

**Background:**

Chronic obstructive pulmonary disease (COPD) is a key public health concern in East Asia. China, Japan, and South Korea represent important regions in East Asia, and the three countries have various similarities in terms of culture, economic development models, and population structure. Therefore, understanding the COPD burden in these three countries is crucial for the prevention and management of COPD in East Asian.

**Methods:**

Age-standardized incidence rate (ASIR), age-standardized prevalence rate (ASPR), age-standardized mortality rate (ASMR), age-standardized disability-adjusted life rate (ASDR), age-period-cohort (APC) analysis, and Bayesian age-period-cohort (BAPC) analysis data of COPD in China, Japan, and South Korea were collected and determined using the GBD (Global Burden of Disease) 2021 database.

**Results:**

China had the highest COPD burden between 1990 and 2021, followed by South Korea, and Japan. The dominant risk factor related to mortality rates and DALYs was environmental/occupational risks in China, whereas behavioral risks in Japan and South Korea were more significant. BAPC predictions indicated that the ASIR, ASPR, and ASDR for COPD in China, Japan, and South Korea would exhibit a downward trend between 2021 and 2031. However, the trend of the age-standardized mortality rate (ASMR) would vary among the three countries.

**Conclusion:**

Under the influence of environmental conditions, the aging population, economic development trends, and the construction of medical security systems, China, Japan, and South Korea share commonalities as well as differences in terms of COPD burden. China’s actions concerning environmental protection have been effective, although reducing smoking rates and alleviating the pollution caused by industrialization are crucial for further reducing the COPD burden in China. Owing to Japan’s environmental protection policies, low smoking rates, and comprehensive social security measures, the COPD burden in Japan is relatively low compared to that in China and South Korea. Owing to the impact of environmental pollution, imperfections in the medical security system, and the influence of South Korea’s handling of related issues after the economic rise of the nation, the COPD burden in South Korea has undergone significant fluctuations.

## 1 Introduction

COPD is a heterogeneous respiratory disease primarily characterized by persistent respiratory symptoms and irreversible airflow obstruction (1). In 2019, COPD was reported as the third most common cause of death globally, following ischemic heart disease and stroke. In 2021, due to the deaths related to the Corona Virus Disease 2019 (COVID-19) pandemic, COPD ranked fourth as the cause of death globally, following ischemic heart disease, COVID-19, and stroke (2). Effective prevention and treatment of COPD is a public health concern worldwide. China, Japan, and South Korea are the three largest economies in East Asia, with their total gross domestic product (GDP) accounting for 22.50% of the global GDP and over 99% of the East Asian GDP, as reported in the 2023 statistics (3, 4). China, Japan, and South Korea also have the largest populations in East Asia, together accounting for 19.8% of the global population and 98% of the East Asian population, according to the 2023 data (5, 6). Moreover, Japan was the first country to enter the phase of an aging society in the year 1970, while China and South Korea entered this phase in 2000. All three countries have been facing serious issues related to population aging, which has prompted these nations to continuously improve their medical security and healthcare systems (7). Moreover, China, Japan, and South Korea belong to the Chinese character cultural circle, with Confucianism as the cultural core, which emphasizes family ethics, hierarchical order, and a culture of “harmony” (8). Economically, these three countries have implemented government-led export-oriented strategies, utilizing industrial policies, investment driven by high savings support, and universal education to increase human capital and achieve compressed industrialization, which forms the “East Asian model of growth” (9). A deep understanding of the COPD burden in China, Japan, and South Korea and deciphering the underlying causes and risk factors are crucial for optimizing the public health policies in the region, rationally allocating health resources, and evaluating the effectiveness of intervention measures to reduce the COPD burden in East Asia.

In this context, this study explored the spatiotemporal heterogeneity, risk factors, and sex differences in the COPD burden in China, Japan, and South Korea. In addition, the future disease burden of COPD in these three countries was predicted. The findings of this study provide a data-driven foundation for the formulation of public health policies in China, Japan, and South Korea, along with data support for determining the high-risk populations and promotion of the rational allocation of medical resources and medical cooperation among the three countries.

## 2 Materials and methods

### 2.1 Materials

The data for this study were collected from the GBD 2021 database developed by the Institute for Health Metrics and Evaluation at the University of Washington, which is one of the largest and most comprehensive global health data resources available to date. This database includes data on 371 diseases and 88 risk factors data 204 countries and regions reported between the years 1990 and 2021. Moreover, this database spans over 80,000 different data sources and provides data on the incidence, prevalence, mortality, DALY, and risk factors associated with related diseases and populations. These data are important for public health policy formulation, resource allocation, and global health research (10).

### 2.2 Methods

This study utilized the 10th edition of the International Classification of Diseases (ICD-10) to classify the diseases under investigation. The data of respondents from Global, China, Japan, and South Korea were selected for the study. Only the respondents with COPD were selected for data collection. The respondents’ gender was set to male, female, or both. The period of 1990–2021 was selected. The following age groups were chosen: “All ages,” “40–44 years,” “45–49 years,” “50–54 years,” “55–59 years,” “60–64 years,” “65–69 years,” “70–74 years,” “75–44 years,” “80–84 years,” “85–89 years,” and “90–94 years” (11). The estimated annual percentage change (EAPC) was calculated as the annual percentage change of a certain indicator in the time series data by fitting a linear regression model. EAPC is usually used to analyze the change trends in annual health indicators. If the EAPC value is greater than 0 and the 95% CI does not go exceed 0, an average annual growth trend; if the EAPC value is less than 0 and the 95% CI does not exceed 0, an average annual decline trend is indicated (12). Data on the COPD incidence rate, prevalence rate, mortality rate, and DALYs stratified by sex and age group in China, Japan, and South Korea for the year 2021 were summarized. The EAPCs and 95% CIs of the ASIR, ASPR, ASMR, and ASDR in China, Japan, and South Korea between the years 1990 and 2021 were calculated. APC analysis is a statistical method used to study changes in disease burden over time to decompose the impact of age, period, and cohort effects on disease incidence rate and mortality (13). In this study, the APC model was used to estimate the net drifts and local drifts of the age model and determine the rate ratio (RR) of the period and cohort effects. In GBD research, RR is obtained by standardizing the disease burden data in GBD and calculating the numerical ratios. RR is commonly used to compare the relative magnitudes of disease burden rates between different populations or time points to determine the changes or differences in disease burden between different populations or periods (14, 15). The equation for the age-standardized rate is as follows (Equation 1):


(1) $$\mathrm{Age}-\text{Standardized Rate}=\frac{\sum_{i=1}^{n}(r_{i}\times w_{i})}{\sum_{i=1}^{n}(w_{i})}\times 100,000$$

*r_i_*: the estimated rate (e.g., mortality, incidence, DALY) for age group i.
*w_i_*: the weight of age group i in the GBD world standard population.
*n*: the number of age groups.

The equation for the RR is as follows (Equation 2):


(2) $$\mathrm{RR}=\frac{\mathrm{Age}-\text{standardized Rate in Group}\;A}{\mathrm{Age}-\text{standardized Rate in Group}\;B}$$

For the identified risk factors, we applied negative binomial regression to estimate the relative risk and subsequently calculated the PAF based on the relative risk. We then used the PAF to assess the disease burden of COPD attributable to various risk factors across different co-occurrence areas (16).

The PAF of the ith risk factor for the *j*th (*j* = 1,2) disease in the *k*th (*k* = 1,2,3) area is calculated as follows (Equation 3) (17):


(3) $${\mathrm{PAF}}_{\mathrm{ijk}}=\frac{\int_{\min}^{\max}\;RR_{\mathrm{ij}}(x)P_{\mathrm{ik}}(x)\mathrm{dx}-\int_{\min}^{\max}\;RR_{\mathrm{ij}}(x)P_{i}^{*}(x)\mathrm{dx}}{\int_{\min}^{\max}\;RR_{\mathrm{ij}}(x)P_{\mathrm{ik}}(x)\mathrm{dx}}$$

The BAPC model is a disease burden prediction model based on the Bayesian statistical framework and mainly used to determine the trends in incidence rates and mortality over time and predict future disease burdens. The BAPC model combines age, period, and cohort effects to comprehensively analyze the dynamic changes in disease burden (18). In this study, BAPC was used to predict the ASIR, ASPR, ASMR, and ASDR for COPD patients from 2021 to 2031. BAPC analysis was performed using integrated nested Laplace approximation (INLA). Smoothing was enforced by assigning independent zero-mean normal priors to the second-order differences of all effects, with the age effect prior specified as follows (Equation 4):


(4) $$f(\alpha |k_{\alpha })\propto k_{\alpha }^{\frac{t-2}{2}}\exp\{-\frac{k_{\alpha }}{2}\sum_{i=3}^{I}\;{\left[(\alpha_{i}-\alpha_{i-1})-(\alpha_{i-1}-\alpha_{i-2})\right]}^{2}\}$$

Considering that we are interested in the incident case counts for age group *α* with a *t* period into the future, the following equation can be applied as follows (Equation 5):


(5) $$\log(Y_{a,p+t})=\mu +\alpha_{a}+\beta_{p+t}+\gamma_{c+t}+\delta_{a,p+t}\;$$

Here, we add an independent random.effect $\delta_{a,p+t}\sim N(0,k_{\delta }^{-1})$ to adjust for overdispersion. Considering the smoothing assumption, the BAPC models assume prior distribution of the period effect as follows (Equation 6) (19):


(6) $$\beta_{p+t}|\beta_{1},\ldots ,\beta_{p},k_{\beta }∼N((1+t)\beta_{p}-t\beta_{p-1},k_{\beta }^{-1}(1+2^{2}+\cdots +t^{2}))\;$$

### 2.3 Statistical analysis

The collected data were organized using Excel 2023 and the tidyverse and reshape2 packages in R 4.4.1. EAPC analysis was performed using Joinpoint 4.9.1.0. The APC Web Tool^1^ was used for APC analysis. BAPC analysis was performed using BAPC along with the INLA package. Data were visualized and analyzed using the GBDR package, the ggpubr package, and the ggplot2 package.

## 3 Results

### 3.1 Disease burden situation

**Figure 1:**
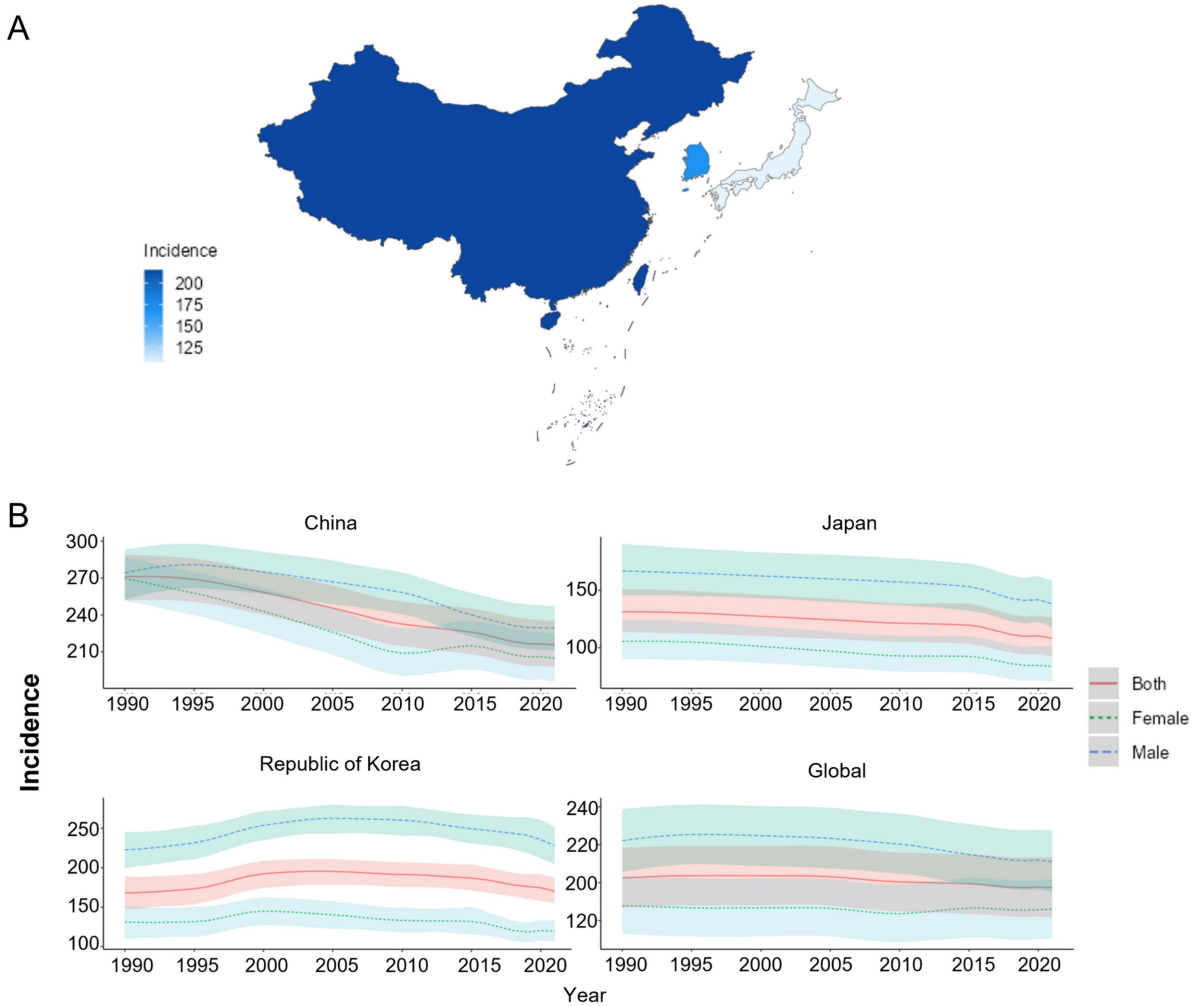
ASIR of COPD patients. **(A)** ASIR for COPD in China, Japan, and South Korea for the year 2021; **(B)** ASIR time trend for COPD. The blue line and the surrounding area represent the prediction curves and the uncertainty intervals for males. The green line and the surrounding area represent the prediction curves and the uncertainty intervals for females. The red line and the surrounding area represent the prediction curves and the uncertainty intervals for both sexes in total.

Among China, Japan, and South Korea, China had the highest ASIR of COPD, with a value of 215.62/100,000 (95% UI: 198.00, 234.90) in the year 2021. South Korea ranked second with a value of 170.31/100,000 (95% UI: 155.14, 186.70), while Japan had the lowest value of 108.24/100,000 (95% UI: 92.19, 126.11). From 1990 to 2021, the ASIR of COPD was greater among males than among females in China, Japan, and South Korea. Additionally, from 1990 to 2021, the ASIR in Japan and South Korea was lower than the global average. Compared to that in 1990, the EAPC of ASIR in South Korea was greater than 0 [00.15 (−0.05, 0.35)], and the EAPC of ASIR in China [−0.84 (−0.88, 0.81)], Japan [−0.56 (−0.63, −0.50)], as well as the global average [−0.11 (−0.13, −0.09)], was less than 0, as shown in Figures 1A,B and Table 1.

**Table 1:** ASIR of COPD and its changing trend.

Country	Age-standardized incidence rate per 100,000 population (95% UI)		EAPCs (95% CI)
	1990	2021	
China	271.22 (251.66, 288.62)	215.62 (198.00, 234.90)	−0.84 (−0.88, −0.81)
Japan	131.36 (113.61, 151.13)	108.24 (92.19, 126.11)	−0.56 (−0.63, −0.50)
South Korea	168.16 (147.82, 188.86)	170.31 (155.14, 186.70)	0.15 (−0.05, 0.35)
Global	202.43 (186.70, 218.25)	197.37 (181.65, 213.42)	−0.11 (−0.13, −0.09)

**Figure 2:**
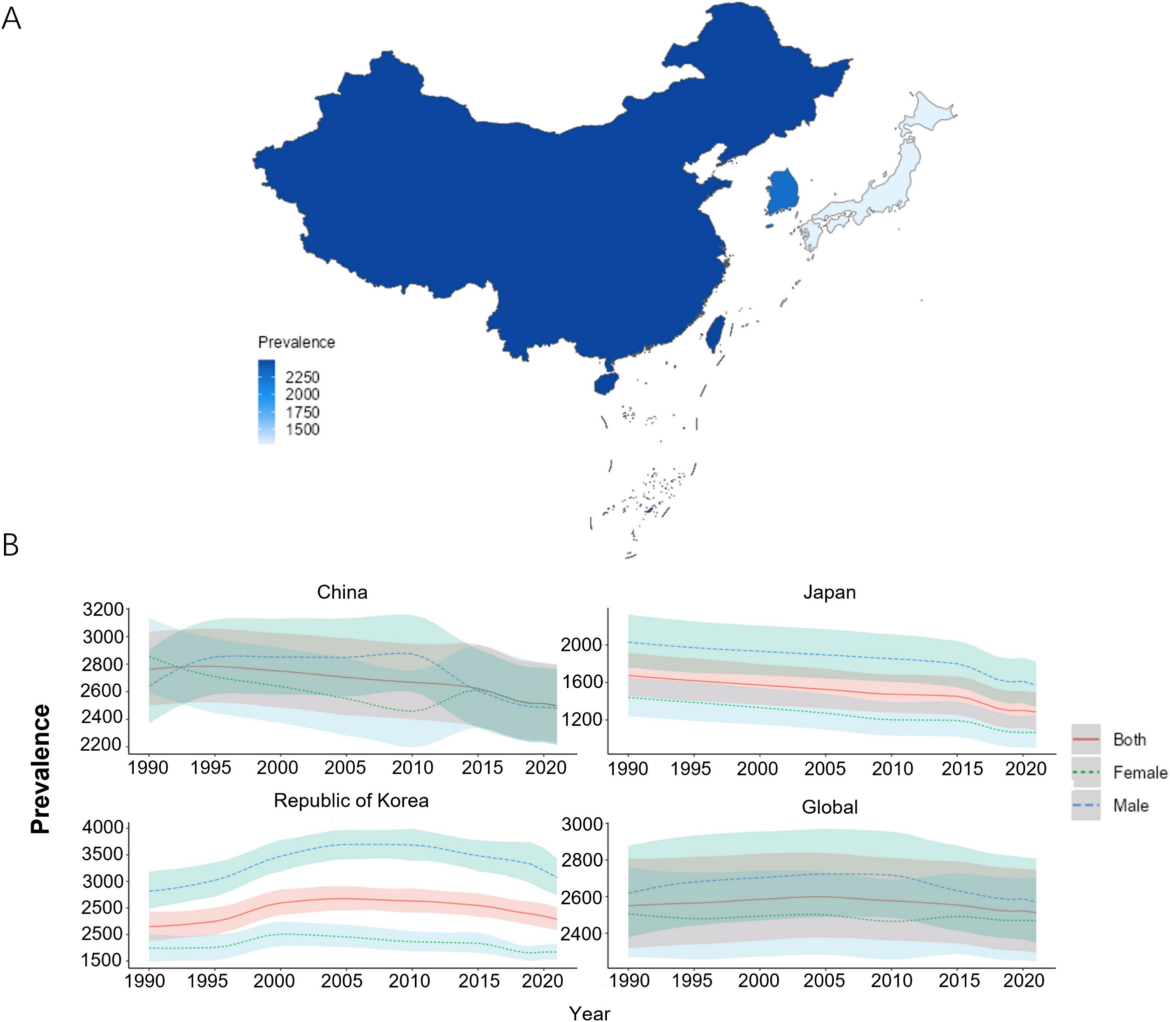
ASPR of COPD patients. **(A)** ASPR for COPD in China, Japan, and South Korea for the year 2021; **(B)** ASPR time trend for COPD. The blue line and the surrounding area represent the prediction curves and the uncertainty intervals for males. The green line and the surrounding area represent the prediction curves and the uncertainty intervals for females. The red line and the surrounding area represent the prediction curves and the uncertainty intervals for both sexes in total.

In 2021, among China, Japan, and South Korea, China had the highest ASPR of COPD, with a value of 2499.35/100,000 (95% UI: 2236.21, 2793.29). South Korea ranked second with an ASPR of 2287.85/100,000 (95% UI: 2091.34, 2510.81). Japan had the lowest ASPR of 1285.53/100,000 (95% UI: 1092.96, 1492.23). From 1990 to 2021, the ASIR of COPD was greater for males than for females in Japan and South Korea. In 1990, the ASPR was lower in Japan [1674.20/100,000 (95% UI: 1455.45, 1916.56)] and South Korea [2149.18/100,000 (95% UI: 1882.65, 2433.37)] compared to the global average [2550.02/100,000 (95% UI: 2318.34, 2806.32)]. In 2021, the ASPR was lower in China [2499.35/100,000 (95% UI: 2236.21, 2793.29)], Japan [1285.53/100,000 (95% UI: 1092.96, 1492.23)], and South Korea [2287.85/100,000 (95% UI: 2091.34, 2510.81)] compared to the global average [2512.86/100,000 (95%UI: 2293.93, 2748.52)]. Compared to 1990, the EAPC for ASPR in South Korea [0.34 (0.08, 0.61)] was greater than 0, whereas the EAPC for ASPR in China [−0.33 (−0.37, 0.29)], Japan [−0.75 (−0.83, 0.67)], and the global average [−0.04 (−0.08, 0.01)] was less than 0, as shown in Figures 2A,B and Table 2.

**Table 2:** ASPR of COPD and its changing trend.

Country	Age-standardized incidence rate per 100,000 population (95% UI)		EAPCs(95% CI)
	1990	2021	
China	2761.81 (2498.94, 3033.60)	2499.35 (2236.21, 2793.29)	−0.33 (−0.37, −0.29)
Japan	1674.20 (1455.45, 1916.56)	1285.53 (1092.96, 1492.23)	−0.75 (−0.83, −0.67)
South Korea	2149.18 (1882.65, 2433.37)	2287.85 (2091.34, 2510.81)	0.34 (0.08, 0.61)
Global	2550.02 (2318.34, 2806.32)	2512.86 (2293.93, 2748.52)	−0.04 (−0.08, −0.01)

**Figure 3:**
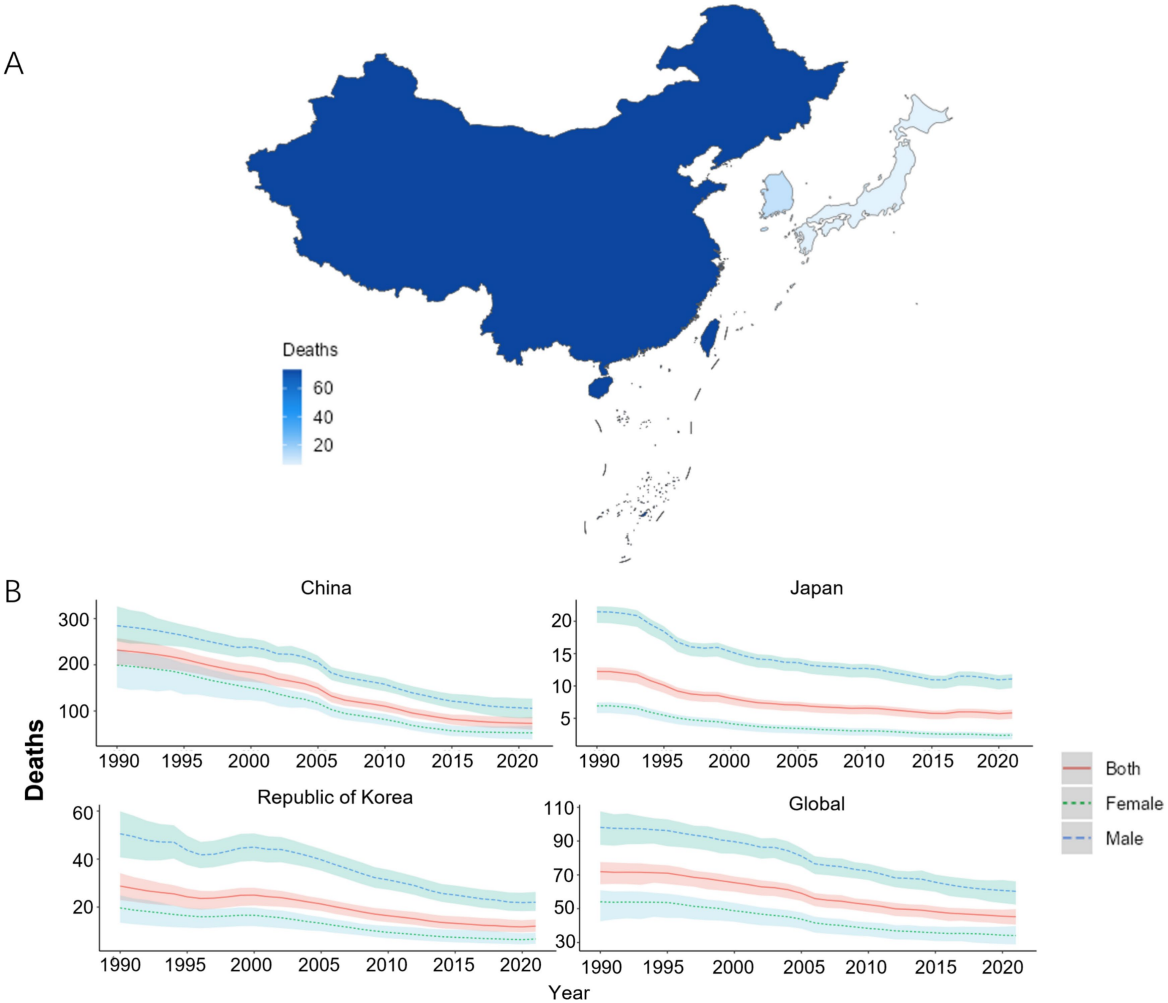
ASMR of COPD patients. **(A)** ASMR for COPD in China, Japan, and South Korea for the year 2021; **(B)** ASMR time trend for COPD. The blue line and the surrounding area represent the prediction curves and the uncertainty intervals for males. The green line and the surrounding area represent the prediction curves and the uncertainty intervals for females. The red line and the surrounding area represent the prediction curves and the uncertainty intervals for both sexes in total.

In 2021, among China, Japan, and South Korea, China had the highest ASMR of COPD, with a value of 73.23/100,000 (95% UI: 59.73, 86.85). South Korea ranked second with a value of 12.03/100,000 (95% UI: 9.74, 14.78). Japan had the lowest value of 5.84/100,000 (95% UI: 4.93, 6.33). From 1990 to 2021, the ASMR for COPD was greater among males than among females in China, Japan, and South Korea. In the 1990–2021 period, the ASMR was lower in Japan and South Korea compared to the global average. Compared to 1990, the EAPC for the ASMR was less than 0 in the case of China [−4.25 (−4.48, −4.02)], Japan [−2.46 (−2.76, −2.16)], South Korea [−3.08 (−3.35, −2.80)], and the global average [−1.75 (−1.84, −1.65)], as shown in Figures 3A,B and Table 3.

**Table 3:** ASMR of COPD and its changing trend.

Country	Age-standardized incidence rate per 100,000 population (95% UI)		EAPCs(95% CI)
	1990	2021	
China	231.78 (198.98, 257.42)	73.23 (59.73, 86.85)	−4.25 (−4.48, −4.02)
Japan	12.24 (10.95, 12.87)	5.84 (4.93, 6.33)	−2.46 (−2.76, −2.16)
South Korea	28.73 (22.89, 34.09)	12.03 (9.74, 14.78)	−3.08 (−3.35, −2.80)
Global	71.92 (64.47, 77.53)	45.22 (40.61, 49.70)	−1.75 (−1.84, −1.65)

**Figure 4:**
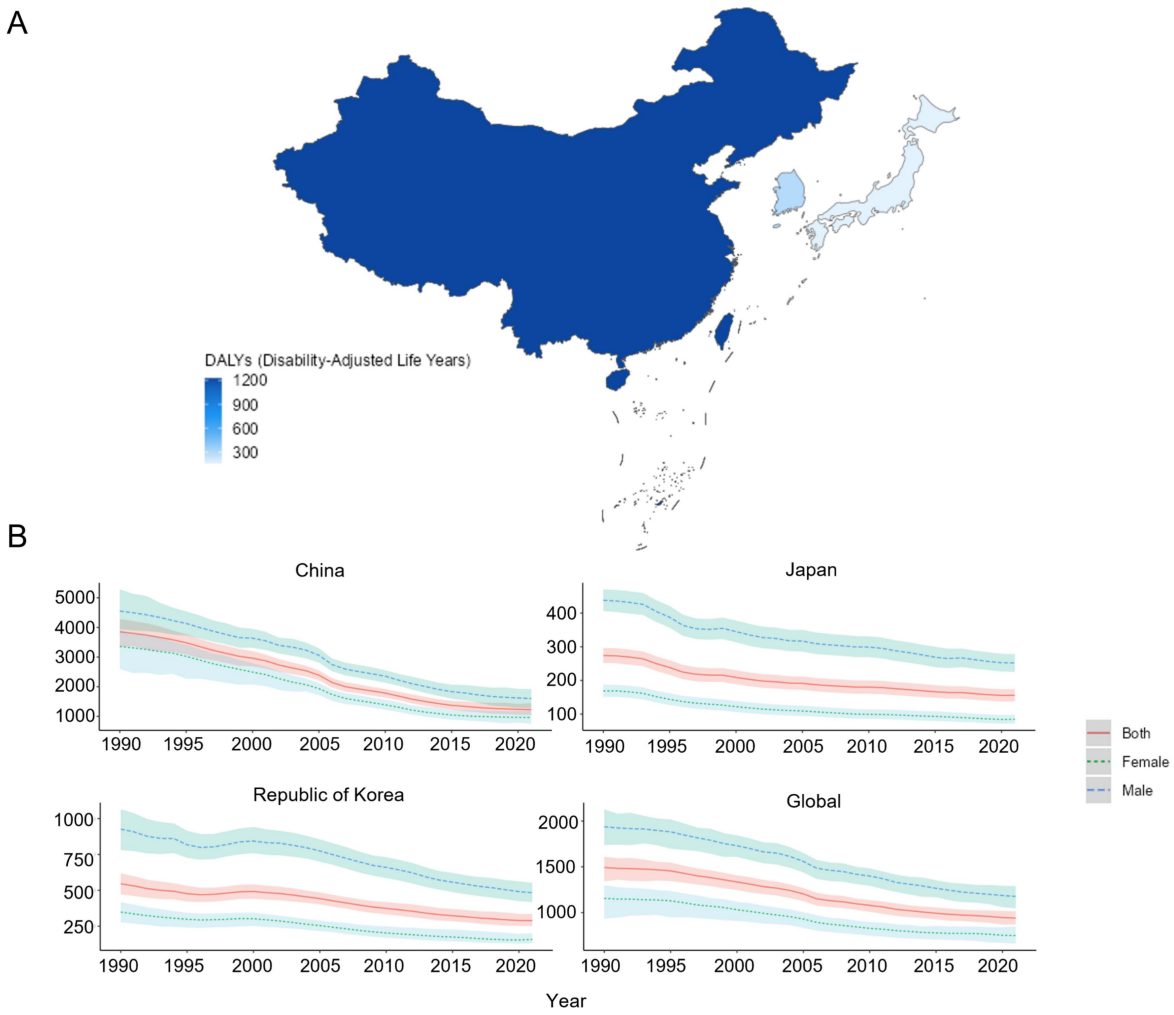
ASDR of COPD patients. **(A)** ASDR for COPD in 2021 of China, Japan, and South Korea for the year 2021; **(B)** ASDR time trend for COPD. The blue line and the surrounding area represent the prediction curves and the uncertainty intervals for males. The green line and the surrounding area represent the prediction curves and the uncertainty intervals for females. The red line and the surrounding area represent the prediction curves and the uncertainty intervals for both sexes in total.

In 2021, among China, Japan, and South Korea, China had the highest ASDR of COPD, with a rate of 1227.66/100,000 (95% UI: 1048.45, 1442.54). South Korea ranked second with a rate of 289.13/100,000 (95% UI: 251.75, 335.70). Japan had the lowest rate of 155.76/100,000 (95% UI: 137.62, 174.26). In the 1990–2021 period, the ASMR of COPD was consistently greater among males than among females in China, Japan, and South Korea. Compared to 1990, the EAPC of ASMR was less than 0 in the case of China [−4.19 (−4.38, −3.99)], Japan [−1.78 (−1.93, −1.63)], South Korea [−2.09 (−2.28, −1.90)], and the global average [−1.71 (−1.79, −1.63)], as shown in Figures 4A,B and Table 4.

**Table 4:** ASDR of COPD and its changing trend.

Country	Age-standardized incidence rate per 100,000 population (95% UI)		EAPCs(95% CI)
	1990	2021	
China	3852.57 (3349.97, 4279.01)	1227.66 (1048.45, 1442.54)	−4.19 (−4.38, −3.99)
Japan	274.06 (251.90, 296.49)	155.76 (137.62, 174.26)	−1.78 (−1.93, −1.63)
South Korea	545.98 (472.24, 617.55)	289.13 (251.75, 335.70)	−2.09 (−2.28, −1.90)
Global	1492.64 (1342.46, 1609.30)	940.66 (871.48, 1014.59)	−1.71 (−1.79, −1.63)

### 3.2 Age and gender differences

**Figure 5:**
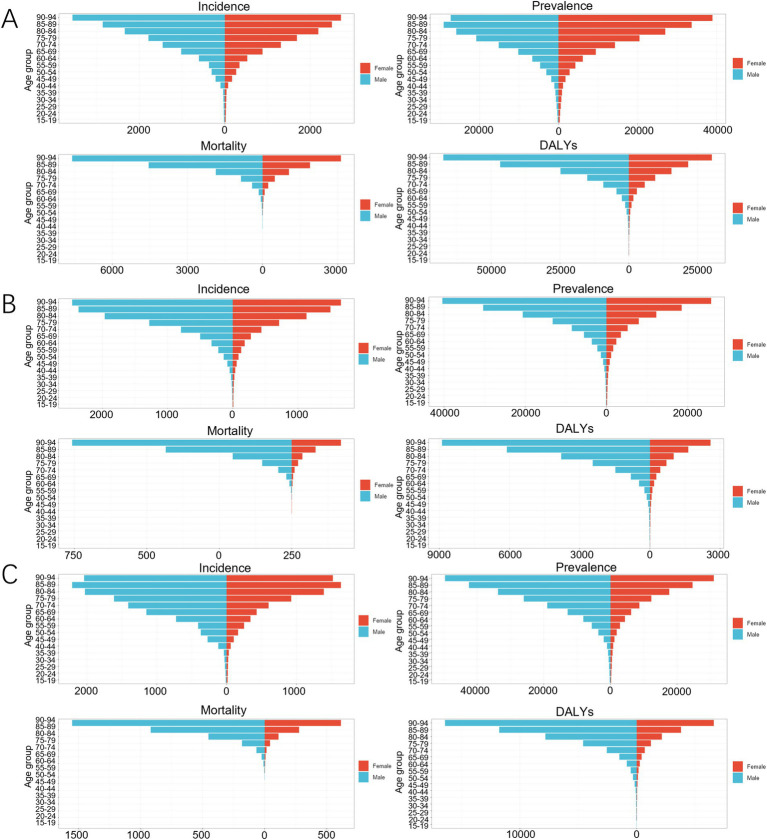
Age-gender comparison of COPD in 2021. **(A)** China; **(B)** Japan; **(C)** South Korea.

In 2021, the incidence rate, prevalence rate, mortality rate, and DALYs of COPD increased with age among Chinese males and females. In the 15–34 year age group, the incidence rate of COPD was higher among females than among males. In the 15–44 year age group and the 80–94 year age group, the prevalence rate of COPD was higher among females than among males. The mortality rate of COPD was lower among females than among males in all age groups. In the 15–34 year age group, the DALY of COPD was greater among females than among males, as shown in Figure 5A.

In 2021, the incidence rate, prevalence rate, mortality rate, and DALYs of COPD increased with age among Japanese males and females. In the 15–34 year age group, the incidence rate of COPD was higher among females than among males. In the 15–44 year age group, the prevalence rate of COPD among females was higher than that among males. The mortality rate and DALY of COPD were lower among females than among males in all age groups, as shown in Figure 5B.

In 2021, the prevalence rate, mortality rate, and DALY of COPD increased with age among Korean males and females. The incidence rate of COPD increased with age in the 15—89 year age range. In the 15–19 year age group, the incidence rate of COPD was higher among females than among males. In the 15–24 year age group, the prevalence rate of COPD was higher among females than among males. The mortality rate and DALY of COPD were lower among females than among males in all age groups, as shown in Figure 5C.

### 3.3 Age-period-cohort model analysis

**Figure 6:**
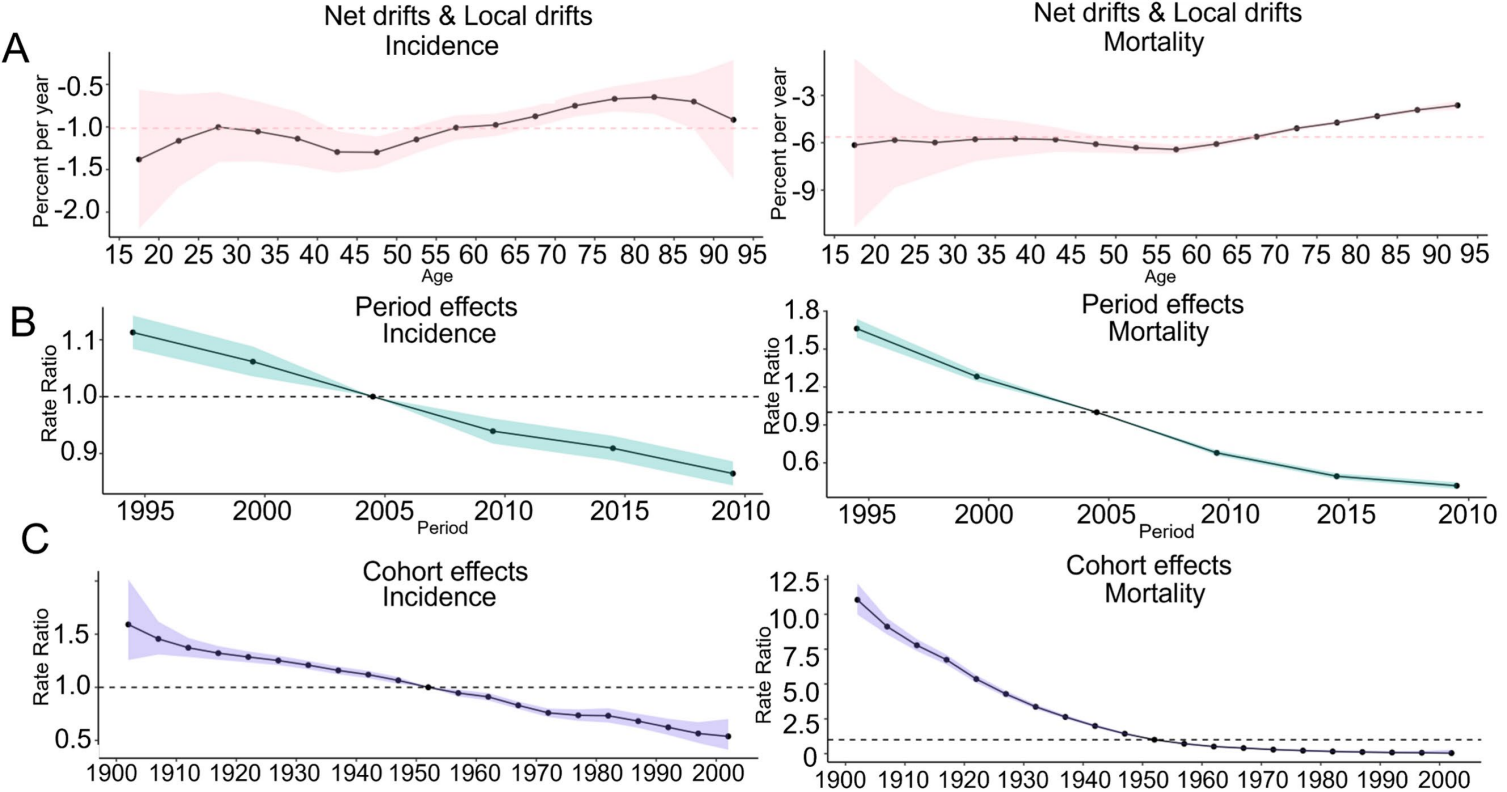
Trends of the APC effect coefficients in China from 1990 to 2021. **(A)** Age effect; **(B)** Period effect; **(C)** Cohort effect. The pink, green, and purple bands in the figure represent the 95% confidence intervals (CIs) of age effects, period effects, and cohort effects, respectively.

In terms of the age effect, the net drifts of the incidence rate (−1.02 [−(−1.12), −(−0.91)]), and mortality rate (−5.63 [−(−6.00), −(−5.26)]) for COPD in China were all less than 0, and the local drifts of the incidence rate and mortality rate for COPD were also less than 0 in all age groups, as shown in Figure 6A. In terms of the period effect, the RR values of the incidence rate and mortality rate for COPD in China exhibited a continuous downward trend from 1990 to 2021, as shown in Figure 6B. In terms of the cohort effect, the RR values of the incidence rate and mortality rate for COPD in China exhibited a continuous downward trend from 1902 to 2002, as shown in Figure 6C.

**Figure 7:**
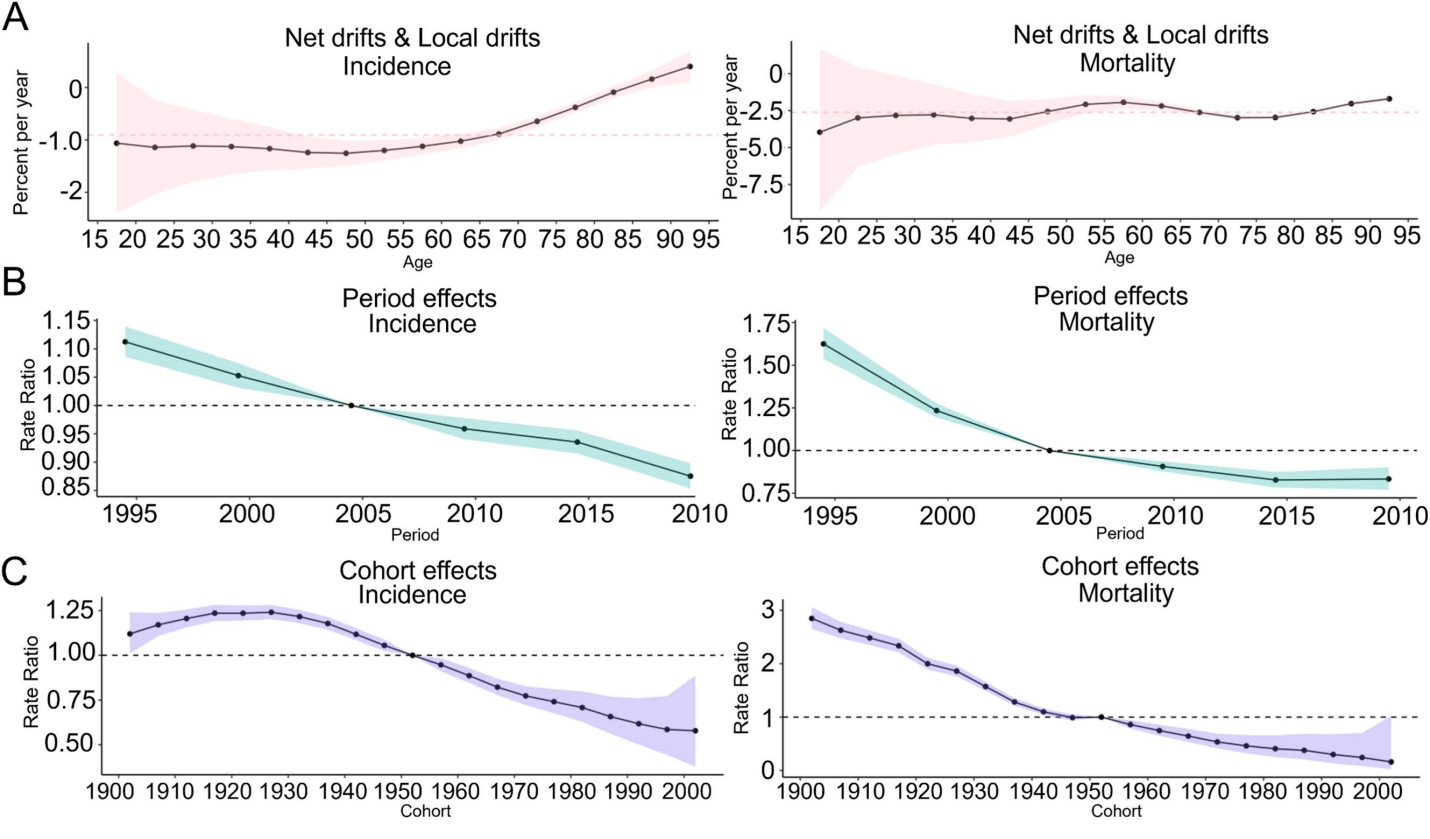
Trends of the APC effect coefficients in Japan from 1990 to 2021. **(A)** Age effect; **(B)** Period effect; **(C)** Cohort effect. The pink, green, and purple bands in the figure represent the 95% confidence intervals (CIs) of age effects, period effects, and cohort effects, respectively.

In terms of the age effect, the net drifts of the incidence rate (−0.91 [−(−1.04), −(−0.77)]) and mortality rate (−2.61 [−(−3.11), −(−2.11)]) of COPD in Japan were less than 0. The local drifts for the incidence rate and mortality rate in the 15–85 years age group were less than 0. However, for those over 85 years of age, the local drifts for the incidence rate were greater than 0, whereas the local drifts for mortality rate were less than 0, as shown in Figure 7A. In terms of the period effect, the RR values of the incidence rate for COPD in Japan exhibited a continuous downward trend from 1990 to 2021. The RR value of the mortality rate exhibited a continuous downward trend from 1990 to 2015, and an upward trend from 2015 to 2021, as shown in Figure 7B. In terms of the cohort effects, the RR of the incidence rate of COPD in Japan exhibited a continuous downward trend from 1927 to 2002. The RR of mortality rates slightly increased from 1947 to 1952 and exhibited a downward trend across all other periods, as shown in Figure 7C.

**Figure 8:**
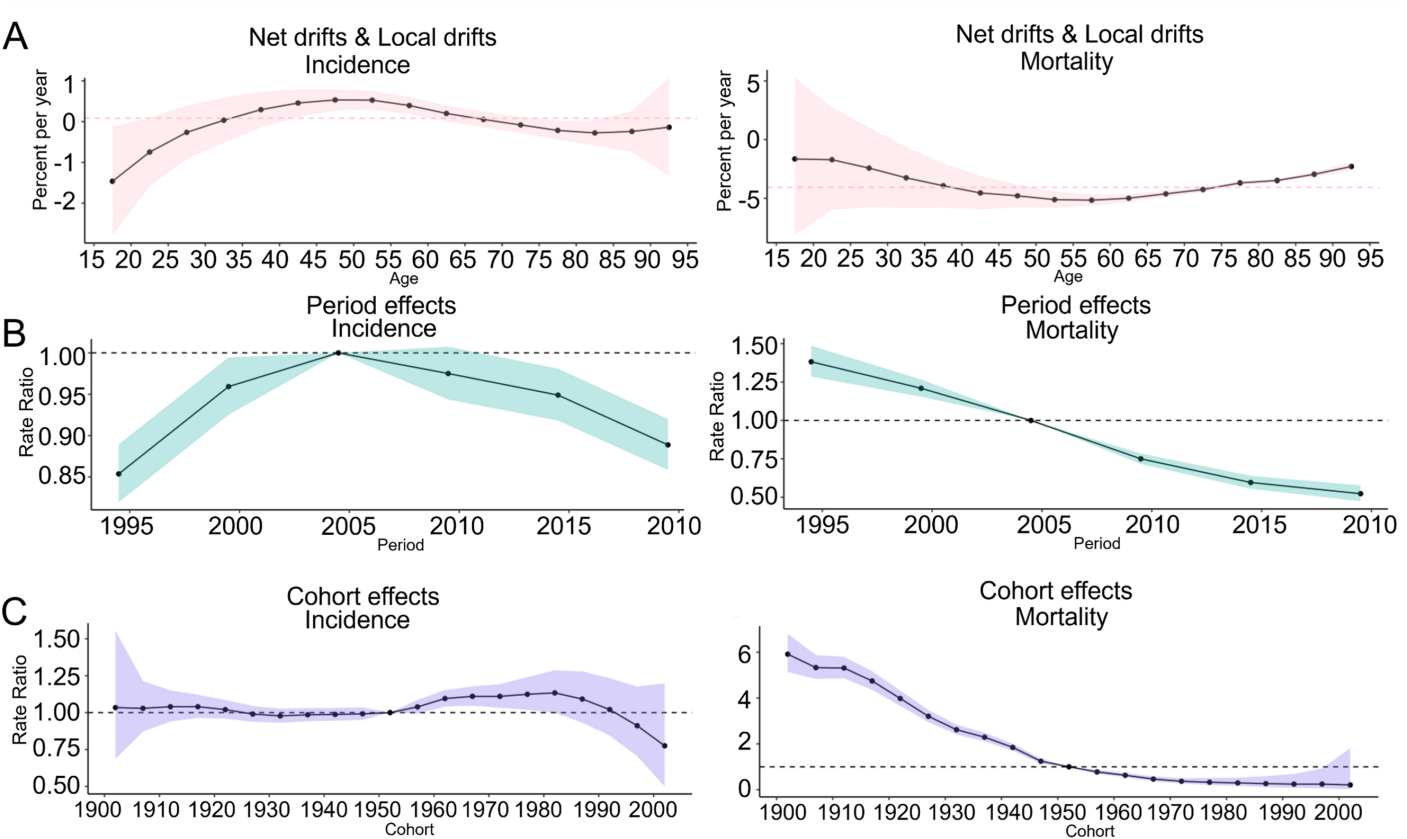
Trends of the APC effect coefficients in South Korea from 1990 to 2021. **(A)** Age effect; **(B)** Period effect; **(C)** Cohort effect. The pink, green, and purple bands in the figure represent the 95% confidence intervals (CIs) of age effects, period effects, and cohort effects, respectively.

In terms of the age effect, the net drifts of the incidence rate of COPD in South Korea [0.08 (−0.08, 0.24)] with a 95% CI, passed through 0, the net drifts of the mortality rate [−4.06 (−4.67, −3.46)] were less than 0, the local drifts of the incidence rate in the 30–70 age group were greater than 0, and the local drifts of the mortality rate and DALY were less than 0 in all age groups, as shown in Figure 8A; In terms of the period effect, the RR values for the incidence rate of COPD in South Korea exhibited a continuous upward trend from 1990 to 2005 and a continuous downward trend from 2005 to 2021. The RR value for the mortality rate exhibited a continuous downward trend from 1990 to 2021, as shown in Figure 8B. In terms of the cohort effects, the RR value of the incidence rate of COPD in South Korea exhibited small fluctuations between 1902 and 1952, exhibiting an increasing trend between 1952 and 1982, followed by a decline from 1982 to 2002. The RR values of the mortality rate and DALY of COPD patients in South Korea demonstrated a continuous downward trend from 1902 to 2002, as shown in Figure 8C.

### 3.4 Risk factors

**Figure 9:**
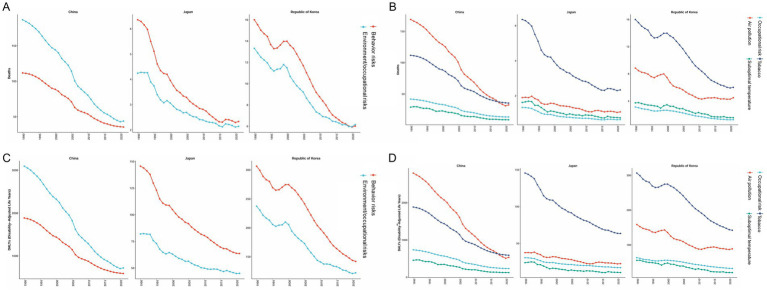
Changes in the PAF of COPD risk factors from 1990 to 2021. **(A)** Level 1 risk factors related to mortality rate; **(B)** Level 2 risk factors related to mortality rate; **(C)** Level 1 risk factors related to DALY; **(D)** Level 2 risk factors related to DALY.

In the period from 1990 to 2021, among the level 1 risk factors for COPD, environmental/occupational risk was the primary risk factor in China. The primary risk factor among the level 2 risk factors for COPD in China shifted from air pollution risk to tobacco risk during the same period. In Japan, the primary risk factor among the level 1 risk factors for COPD in the 1990 to 2021 period was behavioral risk, whereas the primary risk factor among the level 2 risk factors was tobacco risk; In South Korea, the primary risk factor among the level 1 risk factors related to the mortality rate for COPD shifted from behavioral risk to environmental/occupational risk while the primary risk factor among the level 1 risk factors related to DALYs for COPD in South Korea was behavioral risk, while primary risk factor among the level 2 risk factors for COPD was tobacco risk, as shown in Figures 9A–D.

### 3.5 Prediction of disease burden trends

**Figure 10:**
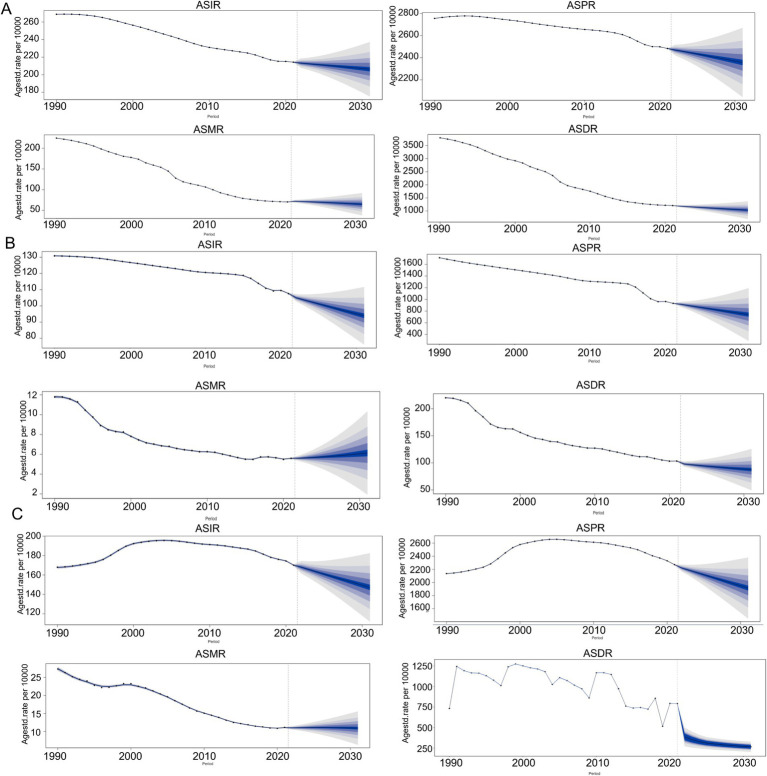
Prediction of COPD burden from 2021 to 2031 period. **(A)** China; **(B)** Japan; **(C)** South Korea. The five different color bands, with a gradient from dark blue to gray, depicted in the figure indicate 95% CI, 80% CI, 70% CI, 60% CI, and 50% CI, respectively.

According to BAPC prediction, the ASIR, ASPR, and ASDR of COPD in China will exhibit a downward trend from 2021 to 2031, whereas the ASMR will exhibit an upward trend from 2021 to 2022, followed by a downward trend from 2022 to 2031, as shown in Figure 10A. The ASIR, ASPR, and ASDR of COPD in Japan will exhibit a downward trend from 2021 to 2031, whereas the ASMR will exhibit an upward trend during the same period, as shown in Figure 10B. In South Korea, the ASIR, ASPR, and ASDR of COPD will exhibit a downward trend from 2021 to 2031, whereas the ASMR will decline from 2021 to 2022, increase from 2022 to 2026, and then decline from 2026 to 2031, as shown in Figure 10C.

## 4 Discussion

### 4.1 China

In the 1990 to 2021 period, China’s ASIR, ASMR, and ASDR for COPD were higher than those of Japan, South Korea, and the global average. China is among the countries with the highest degree of population aging in the world (20). In 2023, the elderly Chinese population aged 65 years and above reached 216.76 million, accounting for 15.4% of the total population of the nation (21). Increasing life expectancy and population aging in China are expected to have a significant impact on the COPD burden of the nation. Elderly individuals have weaker immune and lung functions, and are, therefore, more likely to suffer from chronic diseases and more sensitive to air pollution and chemical products (22, 23). Therefore, the COPD burden of the elderly population is greater than that of the young people, and an increase in the proportion of the elderly population may exacerbate the COPD burden in China. In terms of lifestyle and habits, the smoking rate among Chinese males is as high as 60%, and the smoking rate among Chinese females and adolescents is also rapidly increasing. Furthermore, the willingness of Chinese smokers to quit smoking is quite low, with only 16.1% of the smokers planning or considering quitting smoking (24, 25). The risk factor analysis conducted in this study indicated that the PAF of tobacco was the highest among the level 2 risk factors. Moreover, with rapid industrialization, urbanization, and the development of transportation in China, along with the extensive use of coal and oil, numerous harmful substances have been released into the atmosphere, which has exacerbated the COPD burden in China due to air pollution (26). A Chinese study noted that urbanization and industrialization have increased PM emissions in Chinese cities, and that 64.2% of the 338 PM2.5 samples exceeded National Standard I, 53.0% of the 338 PM10 samples exceeded National Standard II, and 70.7% of urban ambient air quality exceeded National Standard III (27, 28). China is currently a developing country, and its healthcare conditions are relatively weaker than those in Japan and South Korea (29). However, notably, the EAPC of COPD burden in China from 1990 to 2021 was negative for all indicators, and the APC model revealed that the incidence rate and mortality rate of COPD in China continuously declined from 1990 to 2021. Moreover, the COPD burden in China has been consistently alleviated across all age groups. Furthermore, according to the BAPCs, the ASIR, ASPR, and ASDR of COPD in China will exhibit a downward trend from 2021 to 2031, indicating that China has undertaken active and effective measures for the prevention and management of COPD. First, the issue of environmental pollution gradually gained more and more attention in China in these years. In 2007, China proposed the strategic goal of building a resource-saving and environmentally friendly society. Then in 2012, China elevated the construction of an ecological civilization to a new height by explicitly making it one of the goals for the comprehensive construction of a moderately prosperous society and comprehensively deepening reforms and opening up (30). With the implementation of a series of policies directed at environmental protection and industrial energy structure adjustment, China has achieved remarkable results in terms of environmental governance (31). The annual average concentration of PM2.5 in China decreased by 33.3% in 2017 compared to 2013 (27). According to the “2023 China Ecological Environment Status Report,” the average concentration of fine particulate matter (PM) 2.5 in 339 prefecture-level and above cities in China was 30 micrograms per cubic meter, accounting for a cumulative decrease of 28.6% compared to 2016. The proportion of days with good air quality nationwide was 85.5%, which is an increase of 2.4 percentage points compared to 2016 (32). According to the analysis of risk factors in this study, environmental/occupational risk factors remain the main attribution factors for the COPD mortality rate and DAYL, although their proportion significantly decreased from 1990 to 2021. Among the level 2 risk factors, the proportion of the COPD mortality rate and DAYL attributed to air pollution in China exhibited a significant downward trend. In terms of the medical security system, China’s basic medical insurance system covered more than 95% of the population in 2023 (33), and the construction of the “1 + 3 + N” multitier medical security system has narrowed the differences in medical security levels among different regions. The implementation of these measures has increased the accessibility of medical services for patients with COPD (34), thereby alleviating the COPD burden in China to a certain extent.

### 4.2 Japan

In the 1990 to 2021 period, Japan’s ASIR, ASPR, ASMR, and ASDR for COPD were the lowest among the three countries, and Japan’s EAPC for COPD burden was less than 0 for all indicators. According to the BAPC, Japan’s ASIR, ASPR, and ASDR for COPD will all exhibit a downward trend from 2021 to 2031. First, Japan boasts a high level of air quality, with concentrations of PM2.5, PM10, NO2, SO_2_, and CO significantly below the values established in the World Health Organization (WHO) guideline (35). In 2023, Japan’s annual average air quality index (AQI) was 14, owing to which it was listed as the country with the best air quality in Asia (36). According to the analysis of risk factors in this study, the PAF of environmental/occupational risk in Japan remained relatively low from 1990 to 2021. Moreover, the declining trend in smoking rates among Japanese adults may have contributed to the reduction in the COPD burden in Japan. In 1989, the smoking rate was 55.3% among Japanese adult males and 9.4% among females. These rates gradually decreased to 32.1 and 8.5%, respectively, by 2014. According to the analysis of risk factors in this study, the PAF of smoking factors exhibited a continuous downward trend from 1990 to 2021 (37). The healthcare access and quality (HAQ) index is a comprehensive indicator of the accessibility and quality of healthcare and is used in various countries. The index reflects the performance of different countries and regions in terms of providing healthcare services. Japan’s HAQ score was 94, the highest among the three countries, followed closely by South Korea with a score of 90. China, with a score of 78, ranks last among the three nations. Therefore, compared to the other two countries, Japan has superior medical conditions (27, 38).

Although the overall COPD burden in Japan is relatively low, some issues in the prevention and control of COPD remain. First, according to the APC analysis, in terms of the period effect, the RR value of the mortality rate in Japan exhibited a slight upward trend from 2015 to 2021. Moreover, according to the BAPCs predictions, the ASMR of COPD in Japan will increase from 2021 to 2031, which may be related to the rapid advancement of the aging process in Japan. The analysis of the age and gender differences in this study revealed that the incidence rate, prevalence rate, mortality rate, and DALY of COPD in Japan increased with age in both males and females. According to the data released by the Ministry of Internal Affairs and Communications of Japan, as of May 2024, the population aged over 65 years accounted for 29.3% of the total population, which is the proportion in the world (39). Moreover, studies have shown that between 2015 and 2021, the improvement in the chronic disease burden among elderly people in Japan has decelerated (40). The comorbidity of chronic diseases and COPD, as well as Japan’s high aging population structure, may be contributing to an increase in ASMR in Japan in the future. Economic factors may also be contributing to the increase in the ASMR. According to the data from Japan’s Ministry of Internal Affairs and Communications, in 2024, Japan’s consumer price index (CPI) increased by 3% while the wage growth was only 1.9% (41). The decrease in the actual purchasing power may lead low-income groups to postpone physical examinations or reduce medication, thereby exacerbating the COPD burden.

### 4.3 South Korea

In the 1990 to 2021 period, South Korea ranked second among the three countries in terms of the ASIR, ASPR, ASMR, and ASDR of COPD. The EAPC of ASIR, as well as ASPR in South Korea, was greater than 0. According to the APC analysis, in terms of the age effect, the local drifts of the incidence rate in the 30–70 years age group were greater than 0. However, the EAPC of the ASMR as well as ASDR of COPD patients in South Korea, was less than 0. The net drifts of the COPD mortality rate in South Korea were less than 0, and the local drifts of mortality were also less than 0 in all age groups. This may be related to the development trend of the Korean economy. Before the 1970s, South Korea was primarily an agricultural country with a weak industrial foundation, considered a backward economy with an imperfect medical security system. In the period from the 1970s to the mid-1990s, South Korea experienced a prolonged period of rapid economic growth, achieving the globally renowned “Miracle on the Han River” title (42). In this period, South Korea’s steel, shipbuilding, and chemical industries developed rapidly, and this development strengthened the country’s industrial foundation while causing serious environmental pollution. However, during the period of economic take-off, South Korea did not pay sufficient attention to ecological and environmental protection issues, and major pollution incidents such as the “Ulsan Pollution Incident” and the “Nakdong River Pollution Incident” occurred one after another (43). In regard to the medical security system construction, South Korea implemented a policy that prioritized economic development, focused on growth before distribution, and neglected the construction of the medical security system and labor protection (44). The emergence of the “Miracle on the Han River” title was achieved based on low human rights costs and intensive labor (45). Long-term overload has also led to a high incidence of chronic disease in this generation of Koreans. Therefore, under the influence of various factors, the South Koreans who experienced the “Miracle on the Han River” period had a higher incidence and prevalence of COPD compared to the new generation of South Koreans. This finding is also consistent with the cohort effect results of the APC model analysis, which revealed that the increase in the incidence rates was the highest for South Koreans born between 1952 and 1990. Moreover, the generation that experienced the “Miracle on the Han River” gradually entered the aging stage from 1990 to 2021, and the risk of COPD accumulation due to industrialization exploded with increasing age. Consequently, the EAPC of ASIR and ASPR in South Korea was higher than the average levels recorded in Japan, South Korea, and the world overall from 1990 to 2021. With the development of the Korean economy, South Korea has achieved the transformation from an industrial economy to a knowledge-based economy. The medical security system in South Korea has greatly improved, and environmental protection has gradually gained the attention of the government. Around the 1990s, South Korea implemented a policy of parallel medical assistance and medical insurance systems in social medical security, thereby entering the era of universal health insurance (46). In terms of environmental protection, South Korea has formed an environmental legal system that is led by the Basic Act on Environmental Policy (47). Consequently, the COPD burden in South Korea has greatly improved. According to BAPCs prediction, the ASIR, ASPR, and ASDR of COPD in South Korea will decrease from 2021 to 2031. These environmental protection and social security measures have also resulted in an EAPC of less than 0 for the ASMR in South Korea from 1990 to 2021.

## 5 Conclusion

China, Japan, and South Korea belong to the East Asian region and share a similar geography and similar cultural backgrounds, these nations differ in terms of COPD burden. The reasons for this difference may be the different environmental conditions, aging populations, economic development trends, and healthcare system constructions in these countries. This study relies on a single GBD 2021 database, and the results should be analyzed considering several limitations and biases. First, this study relied on aggregated country-level estimates from the GBD database, which enabled comparison between the different countries, although with inherent risks of ecological fallacy. Associations observed at the national level may, therefore, not reflect individual-level exposures or disease mechanisms. Second, the data from the GBD database cannot reflect the gap in disease burden between urban and rural areas in these countries, especially in China, which has vast rural areas and rural populations. Third, the key behavioral risk factors in the GBD database in this study were partially derived from self-report surveys and are subject to recall bias, social desirability bias, and cross-cultural variation in reporting accuracy. Although GBD employs statistical corrections, residual misclassification may persist, which could attenuate true effect sizes for risk-outcome relationships. Fourth, the data in GBD 2021 are derived mainly from statistical reports, research literature, and surveys from various countries. However, the data collection and reporting capabilities of different countries and regions are different, creating variations. Finally, COVID-19 may have affected the collection and accuracy of the data, resulting in deviations in the data to a certain extent.

## Data Availability

The original contributions presented in the study are included in the article/supplementary material, further inquiries can be directed to the corresponding author. All data were obtained from the public open database: Global Health Data Exchange (GHDx) query tool (http://ghdx.healthdata.org/gbd-results-tool).

## Footnotes

^1^ https://analysistools.cancer.gov/apc/
